# A comparison of the FDA TLOVR and FDA Snapshot algorithms based on studies evaluating once-daily vs. twice daily lopinavir/ritonavir (LPV/r) regimens

**DOI:** 10.1186/1758-2652-13-S4-P58

**Published:** 2010-11-08

**Authors:** R Qaqish, J van Wyk, MS King

**Affiliations:** 1 Abbott, Abbott Park, USA; 2 Abbott, Rungis, France

## Purpose

The FDA TLOVR algorithm has been commonly used to assess virologic response to antiretroviral (ARV) regimens. The FDA Snapshot algorithm has been proposed to replace the TLOVR algorithm, as it is simpler and is expected to yield similar results. Multiple studies determined that efficacy of LPV/r dosed once-daily (QD) + nucleoside reverse transcriptase inhibitors (NRTIs) was statistically similar to LPV/r dosed twice-daily (BID) + NRTIs using the FDA TLOVR algorithm. The purpose of the analyses presented here is to compare the TLOVR and Snapshot algorithms in the context of studies evaluating QD vs. BID LPV/r-based regimens.

## Methods

Three studies comparing LPV/r QD + NRTIs vs. LPV/r BID + NRTIs in ARV-naïve (Study 418, n=190; Study 730, n=664) or ARV-experienced (Study 802, n=599) subjects were analyzed. Study results through 48 and 96 weeks were compared using the FDA TLOVR and FDA Snapshot algorithms. The Snapshot algorithm differs from the TLOVR algorithm primarily in its focus only on the visit of interest: a subject is a responder if and only if the subject has an HIV-1 RNA level <50 copies/mL at the visit of interest.

## Results

In the comparison of the FDA TLOVR algorithm to the FDA Snapshot algorithm, 59/1453 (4%) subjects had discordant results (responder by one algorithm but not the other) at week 48, as did 28/854 (3%) at week 96. In each study, LPV/r QD-based regimens provided similar virologic response rates to LPV/r BID-based regimens for each analysis algorithm at each visit (Table 1).

**Table 1:** Percent of subjects with HIV-1 RNA <50 copies/mL using FDA TLOVR and Snapshot algorithms

Week	Analysis Algorithm	Study 418	Study 418	Study 730	Study 730	Study 802	Study 802
		QD (n=115)	BID (n=75)	QD (n=333)	BID (n=331)	QD (n=300)	BID (n=299)

48	TLOVR	71%	65%	78%	77%	55%	52%

48	Snapshot	70%	64%	80%	78%	57%	54%

96	TLOVR	57%	55%	63%	64%	n/a	n/a

96	Snapshot	57%	55%	65%	69%	n/a	n/a

n/a not available, 48-week study

P>0.05 for all QD vs. BID comparisons within each study, analysis algorithm, and timepoint

## Conclusions

The FDA Snapshot analysis is easier to understand, simpler to calculate, and gives similar results compared to the FDA TLOVR algorithm. Efficacy was similar for LPV/r QD-based vs. BID-based regimens in ARV-naïve subjects as well as ARV-experienced subjects, irrespective of timepoint or analysis algorithm.

